# Socioeconomic value of treatments for chronic idiopathic constipation in Japan

**DOI:** 10.1186/s12876-025-04334-8

**Published:** 2025-10-21

**Authors:** Atsushi Nakajima, Aki Unno, Takumi Ota, Ayako Shoji, Tatsuhiro Uenishi, Ataru Igarashi

**Affiliations:** 1 Department of Gastroenterology and Hepatology, Yokohama City University Graduate School of Medicine, 3-9 Fukuura, Kanazawa-Ku, Yokohama, 236-0004 Japan; 2 EA Pharma Co., Ltd., 2-1-1, Irihune, Chuo-Ku, Tokyo, 104-0042 Japan; 3 Mochida Pharmaceutical Co., Ltd., 1-7, Yotsuya, Shinjuku-Ku, Tokyo, 160-0004 Japan; 4 Healthcare Consulting, Inc., 1-8-19, Fujimi, Chiyoda-Ku, Tokyo, 102-0071 Japan; 5 Department of Health Policy and Public Health, Graduate School of Pharmaceutical Sciences, The University of Tokyo, 7-3-1, Hongo, Bunkyo-Ku, Tokyo, 113-0033 Japan

**Keywords:** Chronic idiopathic constipation, Cost-effectiveness, Linaclotide, Lubiprostone, Elobixibat, Socioeconomic burden

## Abstract

**Background:**

Chronic idiopathic constipation (CIC) is a common disorder associated with socioeconomic burden. The aim of this study was to quantify the socioeconomic burden of CIC, including non-traditional value elements, and to compare the cost-effectiveness of 3 treatments for CIC with unique mechanisms of action: elobixibat 10 mg, linaclotide 0.5 mg, and lubiprostone 48 μg.

**Methods:**

We compared medical costs and CIC-specific quality of life (QoL) among patients treated with elobixibat 10 mg, linaclotide 0.5 mg, and lubiprostone 48 μg using a Markov cohort simulation model of 4 mutually exclusive health states (CIC, unimproved, improved, and death) with 4-week cycles and a 2-year time horizon. In addition to costs and QoL scores, which were discounted at 2.0% per year, we considered productivity loss and caregiver burden for patients requiring assistance with daily living and defecation management.

**Results:**

Patients treated with elobixibat 10 mg showed lower total costs and better QoL than those treated with linaclotide 0.5 mg (+ 76,241 Japanese yen [JPY], QoL + 0.034) and lubiprostone 48 μg (JPY + 62,050, QoL + 0.014). The deterministic sensitivity analysis showed that the base-case results were generally robust to changes in most input parameters but were sensitive to the effectiveness of elobixibat and lubiprostone; effectiveness of − 20% for elobixibat and + 20% for lubiprostone resulted in higher costs and poorer QoL for elobixibat 10 mg than lubiprostone 48 μg. The probabilistic sensitivity analysis showed that approximately 72.3% and 64.0% of observations showed better QoL for elobixibat 10 mg than linaclotide 0.5 mg and lubiprostone 48 μg, respectively. Scenario analyses in which no discounting was applied and higher mortality for unimproved patients was assumed yielded similar results to the base-case analysis.

**Conclusions:**

Considering CIC-related medical costs, QoL, productivity loss, and caregiver burden, elobixibat 10 mg is associated with better QoL and lower costs than linaclotide 0.5 mg and lubiprostone 48 μg.

**Trial registration:**

This trial was registered with the UMIN Clinical Trials Registry, registration number UMIN000055903 (registration date: 21 October 2024).

**Supplementary Information:**

The online version contains supplementary material available at 10.1186/s12876-025-04334-8.

## Introduction

Chronic idiopathic constipation (CIC) is a common disorder that physicians treat in clinical practice. CIC is associated with increased risk of all-cause death, cardiovascular disease, and stroke [1] and can cause a variety of issues that contribute to socioeconomic burden, including abdominal pain, sleep disturbance, and productivity loss [2]. However, the impact of CIC on society in Japan is not as well understood as in other countries.

Guidelines for CIC management in Japan have established the role of drugs with unique mechanisms of action (elobixibat, lubiprostone, and linaclotide) for patients whose symptoms do not improve with first-line treatments, such as osmotic and stimulant laxatives, lactulose, and polyethylene glycol [2]. A network meta-analysis (NMA) of randomized clinical trials (RCTs) of drugs found differences in efficacy [3]. However, higher efficacy does not necessarily justify selection because the cost of these drugs vary and their overall costs and benefits have not yet been compared.

A cost-effectiveness study comparing lubiprostone and linaclotide in the United States found that the 2 drugs had similar effectiveness but different in medical costs [4, 5]; however, this analysis included only direct costs. Globally, rising drug prices have driven discussions about how to measure and communicate the full value of treatments beyond medical costs and quality of life (QoL). In 2018, a “value flower” framework was proposed. This framework consists of 12 elements of value [6], including traditional value elements of socioeconomic burden like quality-adjusted life-years, net costs, and productivity loss, and newer concepts, such as family spillover effects and insurance value [7]. Recent studies have expanded the scope of disease burden to include other value elements like caregiver burden, impact on daily life and society, and happiness [8–10]. Given the diverse daily challenges associated with CIC, the burden of this condition likely extends beyond medical costs alone.

The aim of this study was to quantify the socioeconomic burden of CIC in clinical practice using both traditional and non-traditional value elements and to compare the cost-effectiveness of elobixibat, linaclotide, and lubiprostone.

## Methods

### Model overview

**Fig. 1:**
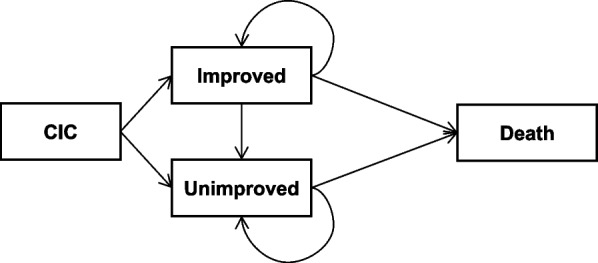
Model structure

This cost-effectiveness analysis compared costs and quality of life (QoL) among patients treated with elobixibat 10 mg, linaclotide 0.5 mg, and lubiprostone 48 μg. We developed a Markov cohort simulation model with 4 mutually exclusive health states (CIC, unimproved, improved, and death), a 4-week cycle, and 2-year time horizon based on previous cost-effectiveness studies on CIC and irritable bowel syndrome with constipation [11–13] (Fig. 1). We chose this simple model to minimize the number of hypothetical parameters and enhance transparency and reproducibility, because of the lack of long-term studies on actual disease transition in patients with CIC undergoing treatment with these drugs in Japan. Costs and QoL were discounted at 2.0% per year, which is the rate recommended for cost-effectiveness analyses in Japan [14]. The mean ± SD age of the initial population was assumed to be 62.9 ± 10.1, based on a survey of patients in Japan with any bowel movement disturbance [15].

Patients with CIC started 1 of the 3 treatments at the start of the model and transitioned to either the unimproved or improved state based on the proportion of patients achieving 3 or more spontaneous bowel movements (SBMs) per week. This threshold was established based on the following: (1) in the Evidence-Based Clinical Guidelines for Chronic Constipation 2023 in Japan, the Japanese Gastroenterological Association defines one of the diagnostic criteria of CIC as “fewer than 3 spontaneous bowel movements per week” [2]; (2) the Cleveland Clinic Constipation Scoring System assigns a score of 0 for fewer than 2 bowel movements per week [16]; and (3) SBM responders are defined as patients with 3 or more SBMs per week and at least 1 additional SBM per week from baseline [17–19].

Patients generally continued their initial treatment and remained in the state they transitioned into after initiation. However, based on expert opinion, 15% of patients in the improved state transitioned to the unimproved state per year irrespective to treatment types, reflecting the likelihood of symptom recurrence despite continued treatment. Otherwise, patients remained in their respective states until death. No patients in the unimproved state transitioned to the improved state.

This trial was registered with the UMIN Clinical Trials Registry (registration number: UMIN000055903; registration date: 21 October 2024). All analyses were based only on secondary information and no human participant was enrolled for this study. The model was programmed in R using a previously validated open-source model [20]. AS developed an initial model, which was updated via an internal validation and quality check by TU.

### Model parameters

Model parameters are shown in Table 1.

**Table 1:** Model parameters

Parameters			Value (per cycle)	Details	Source
Transition probability	Improved (SBM goal achievement)	Elobixibat	0.6012458		Estimated using NMA result
	Improved to unimproved		0.0124	Converted to a 28-day rate	15% per year, based on expert opinion
	mortality		0.0010	Converted to a 28-day rate	12.9 per 1000 people in 2022 [21]
	Risk ratio to Elo	Elobixibat	1.0000		
		linaclotide	0.7155		Estimated using NMA result
		lubiprostone	0.8829		Estimated using NMA result
QoL		Improved state	1.64		Marquis et al. [22]
		Unimproved state	1.88		Marquis et al. [22]
Treatment costs (JPY)	Drug price *	Elobixibat	4,715.2		NHI drug price list
		linaclotide	3,869.6		NHI drug price list
		lubiprostone	5,600.0		NHI drug price list
	Additional treatment in the unimproved state	Disimpaction	333.3	Converted to 28-day costs	NHI Medical Service Fee Points (J022-2): once every 12 weeks
		Enema	1,300.0	Converted to 28-day costs	NHI Medical Service Fee Points (J022): once every 2 weeks
Caregiving costs (JPY)		Improved state	303.3249	% of population aged ≥ 65 years (41.74669) × % of time per day for care in improved state (4.0238095) × % of patients who need care (6.2952) × average wage (286,839.6)	
		Unimproved state	437.9366	% of population aged ≥ 65 years (41.74669) × % of time per day for care in unimproved state (5.8095238) × % of patients who need care (6.2952) × average wage (286,839.6)	
			41.74669%	% of population aged ≥ 65 years	62.9 ± 10.1 [15]
			4.0238095%	% of time per day for care in improved state	16.9 min per work day (7 h) [23]
			5.8095238%	% of time per day for care in unimproved state	24.4 min per work day (7 h) [23]
			6.2952%	% of patients who need care	18.3% (number of people in the population aged 65 or over requiring care) [24] † × 34.4% (proportion requiring defecation management) [25]
			286,839.6	Average wage	311,800 per month [26], converted to 28-day rate
Productivity loss (JPY)		Improved state	34,371.1456	% of population aged < 65 years (58.25331) × % of total work productivity impairment for improved state (20.57) × average wage (286,839.6)	
		Unimproved state	58,783.5149	% of population aged < 65 years (58.25331) × % of total work productivity impairment for unimproved state (35.18) × average wage (286,839.6)	
			58.25331%	% of population aged < 65 years	62.9 ± 10.1 [15]
			20.57%	% of total work productivity impairment for improved state	20.57% (total work productivity impairment in the matched cohort without CIC) [27]
			35.18%	% of total work productivity impairment for unimproved state	35.18% (total work productivity impairment in the cohort diagnosed with CIC) [27]
			286,839.6	Average wage	311,800 per month [26], converted to 28-day rate

*CIC* Chronic idiopathic constipation, *elo* Elobixibat, *JPY* Japanese yen, *NHI* National health insurance, *NMA* Network meta-analysis, *QoL* Quality of life, *SBM* Spontaneous bowel movement

^*^The unit costs for elobixibat 10 mg, linaclotide 0.5 mg, and lubiprostone 48 μg were calculated by multiplying the cost per day from the unit price, listed as of the fiscal year 2024 and 28 days

^†^Calculated by dividing the number of people who needed care services in Japan in 2022 (6,582,416) by the number of people aged 65 years and over in 2022 (36,027,000)

We performed an NMA using the same methods as the previous NMA report, including the same 7 RCTs that reported mean change in SBMs per week for the target drugs (S1 Appendix in Supporting Material), except for those on magnesium oxide and polyethylene glycol [3]. Authors from pharmaceutical companies had no involvement in the NMA. To estimate the proportion of patients achieving 3 or more SBMs per week, we randomly sampled 1000 numbers based on the distribution of the synthesized mean and reported standard deviations (SDs) of the eligible studies and calculated the proportion of numbers greater than 3, following a previous methodology [28]. The resulting proportions were 0.6012458 for elobixibat 10 mg, 0.4302036 for linaclotide 0.5 mg, and 0.5308396 for lubiprostone 48 μg.

QoL was based on the Patient Assessment of Constipation (PAC)-QoL score, a CIC-specific measure that has been used in recent clinical trials and observational studies of patients with CIC to capture disease-specific benefits provided by the target drugs [29–31]. In this measure, higher values correspond to more severe symptoms. Reported values in patients with 3 or more and 0–1 complete evacuations [22] were used for the improved and unimproved states, respectively. These values were accumulated per cycle and divided by 26, the number of cycles in the 2-year time horizon. We also calculated the mean duration in the “improved” health state.

Drug costs were incurred per cycle in both the improved and unimproved states, and additional bowel management costs (i.e., disimpaction every 12 weeks and enema every 2 weeks) were included in the unimproved state. The unit costs for f elobixibat 10 mg, linaclotide 0.5 mg, and lubiprostone 48 μg were calculated based on the cost per day from the unit price, listed as of the fiscal year 2024 (Table 1). Caregiver costs were also considered for patients aged 65 years or over requiring daily living assistance, as this care includes defecation management. These costs were estimated by multiplying (1) the proportion of time per caregiver workday spent on defecation management, (2) the proportion of patients who need daily care including defecation management, and (3) the average wage [26]. For the first value, we used the number of minutes spent on defecation care in patients before (for the unimproved state) and after (for the improved state) treatment with the target drugs [23], converted into proportions of a 7-h day (assumed typical daily working hours for caregivers). The second value was derived by multiplying the proportion of the population aged 65 years and over using any care services [24] and the proportion of that population requiring comprehensive daily care, which includes defecation management [25]. We set the threshold for productivity loss and use of care services as 65 years because employment until 65 years is strongly recommended for employees who want it in Japan as of April 1, 2025.

We additionally considered productivity loss in patients with CIC aged less than 65 years using work impairment data reported by Tomita et al. [27] for patients with (for the unimproved state) and without (for the improved state) CIC, multiplied by the average wage [26].

### Sensitivity analyses

To investigate the uncertainty inherent in the model, deterministic sensitivity analysis (DSA), probabilistic sensitivity analysis (PSA), and scenario analyses were conducted. In the DSA, all input parameters were varied by ± 20% of the base-case value. PSAs were conducted through 10,000 Monte Carlo simulations by sampling from distributions assigned to input parameters (Supplemental Table 2).

Additionally, 6 scenario analyses were conducted. In Scenario 1, we included 2 add-on treatments for patients who remained in the unimproved state for 3 cycles to reflect real clinical practice for such patients. The add-on treatments included stimulant laxatives (senna extract or sennoside, JPY 186.4 per cycle) and osmotic laxatives (magnesium oxide, JPY 365.4 per cycle). We also considered a 4% rate of improvement from the unimproved state, based on rates of 2–6% used in a previous study [12]. In Scenario 2, we used a 0 and 3% discount rates for both costs and QoL, given the short 2-year time horizon. In Scenario 3, we used a mortality rate for the unimproved state 1.19 times higher than that for the improved state, based on previous research demonstrating poorer survival in individuals with symptoms of chronic constipation [32]. In Scenario 4, we extended the time horizon to 5 and 10 years to assess the long-term trend. In Scenario 5, we used alternative overall work impairment rates with less difference between the improved and unimproved states (25.29% and 35.54%, respectively) [33]. In Scenario 6, we excluded caregiving burden, because the data on time spent providing care involved a limited number of nurses.

The statistical analysis software R (version 4.3.0, The R Foundation for Statistical Computing, Vienna, Austria) was used for analysis.

## Results

### Base-case results

Total costs for 2 years of elobixibat 10 mg, linaclotide 0.5 mg, and lubiprostone 48 μg were 1,306,890, 1,383,131, and 1,368,940 Japanese yen (JPY), respectively. QoL scores were 1.697, 1.731, and 1.711, respectively. Patients treated with elobixibat 10 mg showed lower total costs and better QoL scores than those treated with linaclotide 0.5 mg (JPY + 76,241, QoL + 0.034) and lubiprostone 48 μg (JPY + 62,050, QoL + 0.014) (Table 2).

**Table 2:** The result of base-case analysis

Strategy	Costs (JPY)					QoL*		Duration in improved state (mean) (cycles)	
	Medical	Caregiving	Productivityloss	Total	∆ (vs. elo)		∆ (vs. elo)		∆ (vs. elo)
Elobixibat 10 mg	138,266	9,252	1,159,372	1,306,890		1.697		13.273	
Linaclotide 0.5 mg	123,100	9,754	1,250,277	1,383,131	76,241	1.731	0.034	9.476	−3.797
Lubiprostone 48 μg	162,983	9,457	1,196,500	1,368,940	62,050	1.711	0.014	11.722	−1.551

*elo* elobixibat, *JPY* Japanese yen, *QoL* quality of life

*Based on previously reported values of 1.64 (improved state) and 1.88 (unimproved state), which were accumulated every cycle and divided by 26, the number of cycles in the 2-year time horizon

### Sensitivity analysis

The DSA showed that the base-case results were generally robust to changes in most input parameters but were sensitive to the effectiveness of elobixibat and lubiprostone; effectiveness of − 20% for elobixibat and + 20% for lubiprostone resulted in higher costs and poorer QoL for elobixibat 10 mg than lubiprostone 48 μg (Table 3).

**Table 3:** The result of deterministic sensitivity analyses (±20% of the base-case value)

Parameter		Strategy	Lower (−20%)				Upper (+20%)			
			Costs (JPY)		QoL*		Costs (JPY)		QoL*	
				∆ (vs. elo)		∆ (vs. elo)		∆ (vs. elo)		∆ (vs. elo)
Efficacy	elobixibat	Elobixibat 10 mg	1,375,035		1.721		1,238,746		1.673	
		Linaclotide 0.5 mg	1,383,131	8,096	1.731	0.010	1,383,131	144,385	1.731	0.058
		Lubiprostone 48 μg	1,368,940	−6,095	1.711	−0.010	1,368,940	130,194	1.711	0.038
	linaclotide	Elobixibat 10 mg	1,306,890		1.697		1,306,890		1.697	
		Linaclotide 0.5 mg	1,453,026	146,136	1.748	0.051	1,355,732	48,842	1.714	0.017
		Lubiprostone 48 μg	1,368,940	62,050	1.711	0.014	1,368,940	62,050	1.711	0.014
	lubiprostone	Elobixibat 10 mg	1,306,890		1.697		1,306,890		1.697	
		Linaclotide 0.5 mg	1,383,131	76,241	1.731	0.034	1,383,131	76,241	1.731	0.034
		Lubiprostone 48 μg	1,406,889	99,999	1.732	0.035	1,286,526	−20,364	1.690	−0.007
QoL		Elobixibat 10 mg	1,306,890		1.357		1,306,890		2.036	
		Linaclotide 0.5 mg	1,383,131	76,241	1.385	0.028	1,383,131	76,241	2.077	0.041
		Lubiprostone 48 μg	1,368,940	62,050	1.369	0.012	1,368,940	62,050	2.053	0.017
Medical cost		Elobixibat 10 mg	1,302,934		1.697		1,310,848		1.697	
		Linaclotide 0.5 mg	1,377,958	75,024	1.731	0.034	1,388,304	77,456	1.731	0.034
		Lubiprostone 48 μg	1,364,487	61,553	1.711	0.014	1,373,394	62,546	1.711	0.014
Drug cost	elobixibat	Elobixibat 10 mg	1,283,194		1.697		1,330,587		1.697	
		Linaclotide 0.5 mg	1,383,131	99,937	1.731	0.034	1,383,131	52,544	1.731	0.034
		Lubiprostone 48 μg	1,368,940	85,746	1.711	0.014	1,368,940	38,353	1.711	0.014
	linaclotide	Elobixibat 10 mg	1,306,890		1.697		1,306,890		1.697	
		Linaclotide 0.5 mg	1,363,684	56,794	1.731	0.034	1,402,578	95,688	1.731	0.034
		Lubiprostone 48 μg	1,368,940	62,050	1.711	0.014	1,368,940	62,050	1.711	0.014
	lubiprostone	Elobixibat 10 mg	1,306,890		1.697		1,306,890		1.697	
		Linaclotide 0.5 mg	1,383,131	76,241	1.731	0.034	1,383,131	76,241	1.731	0.034
		Lubiprostone 48 μg	1,340,798	33,908	1.711	0.014	1,397,083	90,193	1.711	0.014
Productivity loss		Elobixibat 10 mg	1,075,016		1.697		1,538,765		1.697	
		Linaclotide 0.5 mg	1,133,076	58,060	1.731	0.034	1,633,186	94,421	1.731	0.034
		Lubiprostone 48 μg	1,129,640	54,624	1.711	0.014	1,608,240	69,475	1.711	0.014
Caregiving cost		Elobixibat 10 mg	1,305,040		1.697		1,308,741		1.697	
		Linaclotide 0.5 mg	1,381,180	76,140	1.731	0.034	1,385,082	76,341	1.731	0.034
		Lubiprostone 48 μg	1,367,049	62,009	1.711	0.014	1,370,832	62,091	1.711	0.014

*elo* elobixibat, *JPY* Japanese yen, *QoL* quality of life

*Based on previously reported values of 1.64 (improved state) and 1.88 (unimproved state), which were accumulated every cycle and divided by 26, the number of cycles in the 2-year time horizon

**Fig. 2:**
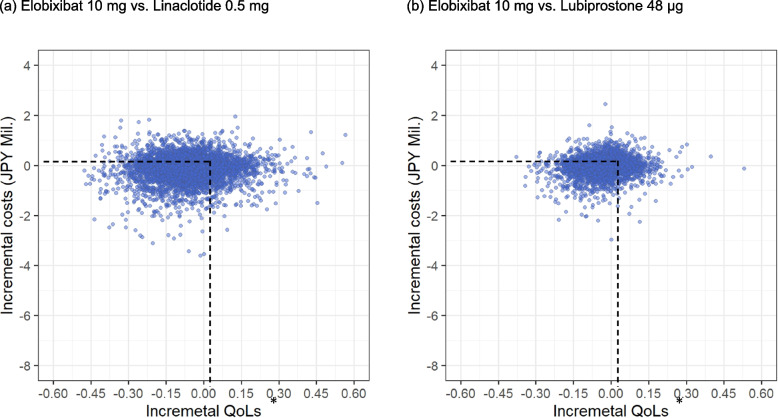
The result of the probabilistic sensitivity analysis

The PSA showed that approximately 72.3% and 64.0% of observations showed better QoL for elobixibat 10 mg than linaclotide 0.5 mg and lubiprostone 48 μg, respectively. Additionally, 57.2% and 51.6% of observations showed lower costs for elobixibat 10 mg than linaclotide 0.5 mg and lubiprostone 48 μg, respectively. Overall, 41.2% and 34.0% of observations showed better QoL and lower costs for elobixibat 10 mg than linaclotide 0.5 mg and lubiprostone 48 μg, respectively (Table 4) (Fig. 2).

**Table 4:** The result of probabilistic sensitivity analysis

Strategy	Costs (JPY)			QoL*		
	Base-case		PSA	Base-case		PSA
	Total	∆ (vs. elo)	95% CrI†		∆ (vs. elo)	95% CI†
Elobixibat 10 mg	1,306,890			1.697		
Linaclotide 0.5 mg	1,383,131	76,241	68,507 – 83,975	1.731	0.034	0.032 - 0.036
Lubiprostone 48 μg	1,368,940	62,050	57,020 – 67,080	1.711	0.014	0.013 - 0.015

*elo* elobixibat, *JPY* Japanese yen, *QoL* quality of life, *PSA* probabilistic sensitivity analysis

*Based on previously reported values of 1.64 (improved state) and 1.88 (unimproved state), which were accumulated every cycle and divided by 26, the number of cycles in the 2-year time horizon

†Derived through 10000 Monte Carlo simulations by sampling from distributions assigned to input parameters

#### Scenario analyses

In Scenario 1, in which patients who remained in the unimproved state for 3 cycles received additional stimulant or osmotic laxatives, total costs increased for all target drugs. Elobixibat was associated with lower total costs (Table 5) and better QoL (unchanged from base-case analysis) than the other 2 drugs, but the difference in total costs decreased (Table 5).

**Table 5:** The result of scenario analysis: add-on treatments in the unimproved state (additional costs and transition from the unimproved to improved state)

Strategy	Total costs (JPY)	∆ (vs. elo)	QoL*	∆ (vs. elo)	Add-on costs (stimulant) (JPY)	∆ (vs. elo)	Add-on costs (osmotic) (JPY)	∆ (vs. elo)
Elobixibat 10 mg	1,210,359		1.663		1,410		2,764	
Linaclotide 0.5 mg	1,254,640	44,281	1.686	0.023	1,847	44,718	3,621	45,138
Lubiprostone 48 μg	1,259,356	48,997	1.672	0.009	1,589	49,176	3,114	49,347

*elo* Elobixibat, *JPY* Japanese yen, *QoL* Quality of life

^*^Based on previously reported values of 1.64 (improved state) and 1.88 (unimproved state), which were accumulated every cycle and divided by 26, 65, and 130, the number of cycles in the 2-, 5-, and 10-year time horizon

Scenario 2, in which no discounting and 3% discount rate were applied for costs and QoL, and Scenario 3, in which higher mortality was assumed for the unimproved state, yielded similar results to the base-case analysis (Tables 6 and 7).

**Table 6:** The result of scenario analysis: other discount rates

	Strategy	Costs (JPY)					QoL*	
		Medical	Caregiving	Productivity loss	Total	∆ (vs. elo)		∆ (vs. elo)
0%	Elobixibat 10 mg	141,195	9,449	1,184,084	1,334,728		1.733	
	Linaclotide 0.5 mg	125,704	9,960	1,276,798	1,412,462	77,734	1.768	0.035
	Lubiprostone 48 μg	166,428	9,658	1,221,951	1,398,037	63,309	1.747	0.014
3%	Elobixibat 10 mg	136,813	9,155	1,147,112	1,293,080		1.679	
	Linaclotide 0.5 mg	121,809	9,651	1,237,119	1,368,579	75,499	1.713	0.034
	Lubiprostone 48 μg	161,274	9,358	1,183,874	1,354,506	61,426	1.693	0.014

*elo* Elobixibat, *JPY* Japanese yen, *QoL* Quality of life

^*^Based on previously reported values of 1.64 (improved state) and 1.88 (unimproved state), which were accumulated every cycle and divided by 26, 65, and 130, the number of cycles in the 2-, 5-, and 10-year time horizon

**Table 7:** The result of scenario analysis: higher mortality in the unimproved state

Strategy	Costs (JPY)					QoL*	
	Medical	Caregiving	Productivity loss	Total	∆ (vs. elo)		∆ (vs. elo)
Elobixibat 10 mg	138,098	9,241	1,157,816	1,305,155		1.695	
Linaclotide 0.5 mg	122,905	9,738	1,248,185	1,380,828	75,673	1.729	0.034
Lubiprostone 48 μg	162,765	9,444	1,194,725	1,366,934	61,779	1.709	0.014

*elo* Elobixibat, *JPY* Japanese yen, *QoL* Quality of life

^*^Based on previously reported values of 1.64 (improved state) and 1.88 (unimproved state), which were accumulated every cycle and divided by 26, 65, and 130, the number of cycles in the 2-, 5-, and 10-year time horizon

Scenario 4, in which the time horizon was extended, yielded similar results to the base-case analysis (Table 8), but the difference in QoL between drugs was reduced because a greater proportion of patients remained in the unimproved state over time (Supplementary Figure S4).

**Table 8:** The result of scenario analysis: longer time horizon

Scenario	Strategy	Costs (JPY)					QoL*	
		Medical	Caregiving	Productivity loss	Total	∆ (vs. elo)		∆ (vs. elo)
5-year	Elobixibat 10 mg	338,911	22,828	2,905,081	3,266,820		1.638	
	Linaclotide 0.5 mg	300,035	23,795	3,080,310	3,404,140	137,320	1.665	0.027
	Lubiprostone 48 μg	396,644	23,223	2,976,650	3,396,517	129,697	1.649	0.011
10-year	Elobixibat 10 mg	645,522	43,772	5,656,396	6,345,690		1.535	
	Linaclotide 0.5 mg	568,166	45,102	5,897,584	6,510,852	165,162	1.553	0.018
	Lubiprostone 48 μg	749,940	44,315	5,754,904	6,549,159	203,469	1.543	0.008

*elo* Elobixibat, *JPY* Japanese yen, *QoL* Quality of life

^*^Based on previously reported values of 1.64 (improved state) and 1.88 (unimproved state), which were accumulated every cycle and divided by 26, 65, and 130, the number of cycles in the 2-, 5-, and 10-year time horizon

In Scenario 5, assuming overall work impairment rates with less margin between the improved and unimproved states [33], and Scenario 6, excluding caregiving burden, the difference in total costs between drugs decreased, but overall results were similar (Tables 9 and 10).

**Table 9:** The result of scenario analysis: no caregiving burden

Strategy	Costs (JPY)					QoL*	
	Medical	Caregiving	Productivity loss	Total	∆ (vs. elo)		∆ (vs. elo)
Elobixibat 10 mg	138,266	0	1,159,372	1,297,638		1.697	
Linaclotide 0.5 mg	123,100	0	1,250,277	1,373,377	75,739	1.731	0.034
Lubiprostone 48 μg	162,983	0	1,196,500	1,359,483	61,845	1.711	0.014

*elo* Elobixibat, *JPY* Japanese yen, *QoL* Quality of life

^*^Based on previously reported values of 1.64 (improved state) and 1.88 (unimproved state), which were accumulated every cycle and divided by 26, 65, and 130, the number of cycles in the 2-, 5-, and 10-year time horizon

**Table 10:** The result of scenario analysis: higher overall impairment in the improved state

Strategy	Costs (JPY)					QoL*	
	Medical	Caregiving	Productivity loss	Total	∆ (vs. elo)	QoL*	∆ (vs. elo)
Elobixibat 10 mg	138,266	9,252	1,269,301	1,416,819		1.697	
Linaclotide 0.5 mg	123,100	9,754	1,333,078	1,465,932	49,113	1.731	0.034
Lubiprostone 48 μg	162,983	9,457	1,295,349	1,467,789	50,970	1.711	0.014

*elo* Elobixibat, *JPY* Japanese yen, *QoL* Quality of life

^*^Based on previously reported values of 1.64 (improved state) and 1.88 (unimproved state), which were accumulated every cycle and divided by 26, 65, and 130, the number of cycles in the 2-, 5-, and 10-year time horizon

## Discussion

We investigated the cost-effectiveness of 3 CIC treatments with unique mechanisms of action and found that elobixibat 10 mg was associated with lower costs and higher QoL over 2 years than linaclotide 0.5 mg and lubiprostone 48 μg. This is the first study comparing the cost-effectiveness of these 3 drugs as treatments for CIC. In addition to traditional value elements such as medical costs, QoL, and productivity loss, we also included caregiving costs as an additional value element. However, the difference in QoL between drugs was smaller than the change reported in patients who self-rated their CIC as “minimally improved” (− 0.55) [22].

The results of the DSA showed that the parameter with the highest impact on the base-case results was drug effectiveness. Although the effectiveness values were derived from an NMA of RCT data, we applied a ± 20% variation (sufficiently wider variability than expected in clinical settings) to robustly assess the impact of uncertainty. However, because the settings in RCTs may differ from actual clinical conditions, and given the uncertainty in efficacy suggested in the previous NMA study [3], ongoing evaluation of real-world efficacy is important to refine these cost-effectiveness estimates further. The results of the PSA suggest that elobixibat was associated with better QoL but no superiority in total costs.

The model used in this study was based on previously published simple models for CIC [11–13]. To validate its applicability to real-world clinical conditions, we performed 6 scenario analyses to account for factors such as add-on treatment for patients in the unimproved state, different discount and mortality rates, longer time horizons, and a smaller difference in productivity loss between the improved and unimproved states and found that all of them had a limited impact on the base-case results, indicating the robustness of our results.

Previous cost-effectiveness studies in the United States have examined linaclotide and lubiprostone. One study estimated monthly medical costs of 727 U.S. dollars (USD) for linaclotide and USD 737 for lubiprostone (equivalent to 2-year costs of about JPY 2,617,200 and 2,653,200, respectively [1 USD = 150 JPY]) [5], while another study of patients treated with linaclotide reported medical costs of USD 33,453 (JPY 5,017,950) and productivity loss of USD 36,972 (JPY 5,545,800) [34]. However, given that drug costs are significantly higher in the US than in Japan (linaclotide USD 523 [35] vs 28.0, lubiprostone USD 374 [35] vs. 40.6), these results are not directly applicable to the Japanese setting. Nonetheless, both the US and Japanese studies indicate that the cost of productivity loss exceeds that of medical treatment. Recent surveys of Japanese patients with CIC reported annual productivity losses of JPY 1.218 million and 1.343 million [27, 36], which are comparable to our results if all patients were assumed at work (JPY 0.995–1.073 million per year), particularly considering that more than half of patients remained in the improved state throughout the study period.

In Japanese clinical guidelines, all 3 study drugs are recommended equally [2], with no specific recommendations differentiating them. However, the drugs have slightly different unit prices (Table 1) and effectiveness, as reported in a previous study [3]. Recently, there has been increasing focus on CIC-related symptoms, as CIC involves various symptoms that do not necessarily impose large medical costs but can significantly impact daily life and work productivity [36]. Additionally, some symptoms that exacerbate CIC can be improved by new drugs [37]. The latest Japanese guidelines recommend considering symptoms at diagnosis [2]. Therefore, when selecting treatments for CIC, it is important to consider not only therapeutic effects, such as SBMs, but also other benefits, including improved QoL, reduced productivity loss, and reduced caregiver burden. In the present study, we considered an expanded socioeconomic burden beyond traditional value elements and found that elobixibat 10 mg was likely more cost-effective than the comparators. The cost-effectiveness of the subject drugs may change when considering their unique mechanisms of action (elobixibat: water secretion, promotion of colonic peristalsis, and restoration of the urge to defecate; linaclotide: activation of guanylate cyclase C, secretion of chloride ion and bicarbonate ion into the intestinal lumen, and concomitant fluid secretion; lubiprostone: activation of the type 2 chloride ion channel, promotion of the secretion of electrolytes and fluid into the intestinal tract), which are thought to influence CIC-related symptoms [38]. Patients with CIC are often unaware of the broader socioeconomic burdens associated with their condition, but providing this information along with data on clinical efficacy may help guide treatment selection and encourage adherence through communication between patients and physicians. We focused on CIC-specific clinical values, which is narrow in terms of policy implications, but our results may have broader implications for therapeutic decision-making, similarly to other therapeutic areas where numerous treatment options exist for a given condition.

### Limitations of this study

This study has 2 important limitations. First, the base-case results showed some uncertainty, primarily driven by drug efficacy. We believe NMA is a reliable method to quantitatively synthesize efficacy data from RCTs, but the observed heterogeneity may contribute to uncertainty in our results (S1 Appendix). If real-world efficacy differs from RCT data, further research using real-world evidence will be necessary to refine cost-effectiveness estimates and strengthen generalizability. Second, although we considered an expanded set of cost-effectiveness elements beyond traditional ones, our analysis was limited to elements with quantifiable data, and other elements may affect cost-effectiveness. If data on these elements in Japan becomes available, future studies could more comprehensively evaluate the value of CIC treatments.

Other limitations are as follows: (1) We developed the simple and versatile model to enhance transparency and reproducibility, but it has limited ability to capture state- or drug-specific transitions, such as tolerability and adverse effects commonly seen in clinical practice. If these factors are considered, the results might differ; for example, a recent study reported a difference in the number needed to harm between drugs [29]. (2) We used a disease-specific measure of QoL, which has the advantage of representing disease-specific benefits and issues, but for comprehensive comparison between diseases in the context of health technology assessments, mapping to generic health utility measures is required. (3) We assumed a 4% improvement rate from the use of add-on stimulant or osmotic laxatives for patients who remained in the unimproved state, although no data were available to support it. (4) Our results may not be applicable to populations with substantially different responses to the target drugs than the Japanese population. (5) The results of this study may be influenced by factors such as the use of generic drugs.

## Conclusions

Considering the medical costs, QoL, productivity loss, and caregiver burden associated with CIC treatments, elobixibat 10 mg was associated with better QoL and lower costs than linaclotide 0.5 mg and lubiprostone 48 μg.

## Supplementary Information

Supplementary Material 1: Supporting_material_Socioeconomic value for CIC.

## Data Availability

All data generated or analyzed during this study are available in published RCTs, systematic reviews, and other relevant literature, as shown in Table 1 and the Supporting Material. Our model was developed from a previously published open-source algorithm [20].

## Abbreviations

CIC: Chronic idiopathic constipation
DSA: Deterministic sensitivity analysis
NMA: Network meta-analysis
PSA: Probabilistic sensitivity analysis
QoL: Quality of life
RCT: Randomised clinical trial
SBM: Spontaneous bowel movement
JPY: Japanese yen
PAC: Patient Assessment of Constipation
USD: U.S. dollars
