# Supplementary material for: Socioeconomic value of treatments for chronic idiopathic constipation in Japan
Source: BMC Gastroenterol. 2025 Oct 21;25:748. doi: 10.1186/s12876-025-04334-8 (PMC12542378; doi:10.1186/s12876-025-04334-8)
Supplement: Supplementary file 1 — Supplementary Material 1: Supporting_material_Socioeconomic value for CIC. [file 12876_2025_4334_MOESM1_ESM.docx]

**S1 Appendix**

**Network Meta-Analysis**

Eligible studies had to meet the same criteria as a previous study [1] but were limited to the target drugs in the present study, as follows: (1) the study was a published randomized controlled trial (RCT) with at least 1-week follow-up; (2) the study population comprised Japanese adults (aged ≥ 18 years) with chronic idiopathic constipation (CIC) diagnosed according to the Rome II, III, or IV criteria or variations of these; (3) the studied CIC medications were elobixibat 10 mg, linaclotide 0.5 mg, and lubiprostone 48 μg, and the study compared their dosage forms with each other or with placebo; (4) the study evaluated relevant bowel function measurements, including spontaneous bowel movements (SBMs); (5) the publication was written in English or Japanese; and (6) the study was published between 1 January 2010 and 5 February 2024.

According to the criteria, we conducted a systematic search in MEDLINE via PubMed on 5 February, 2024 using search terms shown in Table S1.

**Table S1. Search strategies.**

| # | Query | Results |
| --- | --- | --- |
| 1 | Constipation[MeSH Terms] | 16,437 |
| 2 | "chronic constipation"[tw] OR "chronic idiopathic constipation"[tw] | 3,815 |
| 3 | #1 OR #2 | 17,737 |
| 4 | AJG555[tiab] OR A3309[tiab] OR DB1248[tiab] OR elobixibat[tiab] OR Goofice[tiab] OR "IBAT inhibitor"[tiab] OR ASP0456[tiab] OR linaclotide[tiab] OR Linzess[tiab] OR constella[tiab] OR lubiprostone[tiab] OR Amitiza[tiab] OR Macrogol[tiab] OR MOVICOL[tiab] OR "Polyethylene Glycol"[tiab] OR "Magnesium oxide"[tiab] OR "SK?1202"[tiab] OR "crystalline lactulose preparation"[tiab] OR "crystallized lactulose preparation"[tiab] | 34,715 |
| 5 | (randomized controlled trial [pt] OR controlled clinical trial [pt] OR randomized [tiab] OR placebo [tiab] OR clinical trials as topic [mesh: noexp] OR randomly [tiab] OR trial [ti]) NOT (animals [mh] NOT humans [mh]) | 1,548,007 |
| 6 | non-Randomized Controlled Trials as Topic[mh] OR Controlled clinical trial [pt] OR Controlled Clinical Trials as Topic [mh] OR comparative study[pt] OR Clinical trial[pt] OR clinical trial [mh] OR Clinical trials as topic[mh] OR cross-over studies [mh] | 2,981,226 |
| 7 | meta-analysis [pt] OR meta-analysis [mh] OR "meta-analysis" [All] or (systematic[sb] AND review[pt]) OR "systematic review" [All] | 469,891 |
| 8 | #5 OR #6 OR #7 | 3,766,379 |
| 9 | #3 AND #4 AND #8 | 399 |

One reviewer (AS) reviewed the titles and abstracts and excluded studies that did not meet the inclusion criteria. The full texts of the remaining articles were then obtained, and the same reviewer evaluated whether the studies met the inclusion criteria. Discrepancies between the included articles and those in the previous report [1] were confirmed by a second reviewer (AU) and resolved through discussions with a third reviewer (TO). Because no articles published since the search date of the previous study [1] met the present inclusion criteria, quality assessment was not performed for the present study.


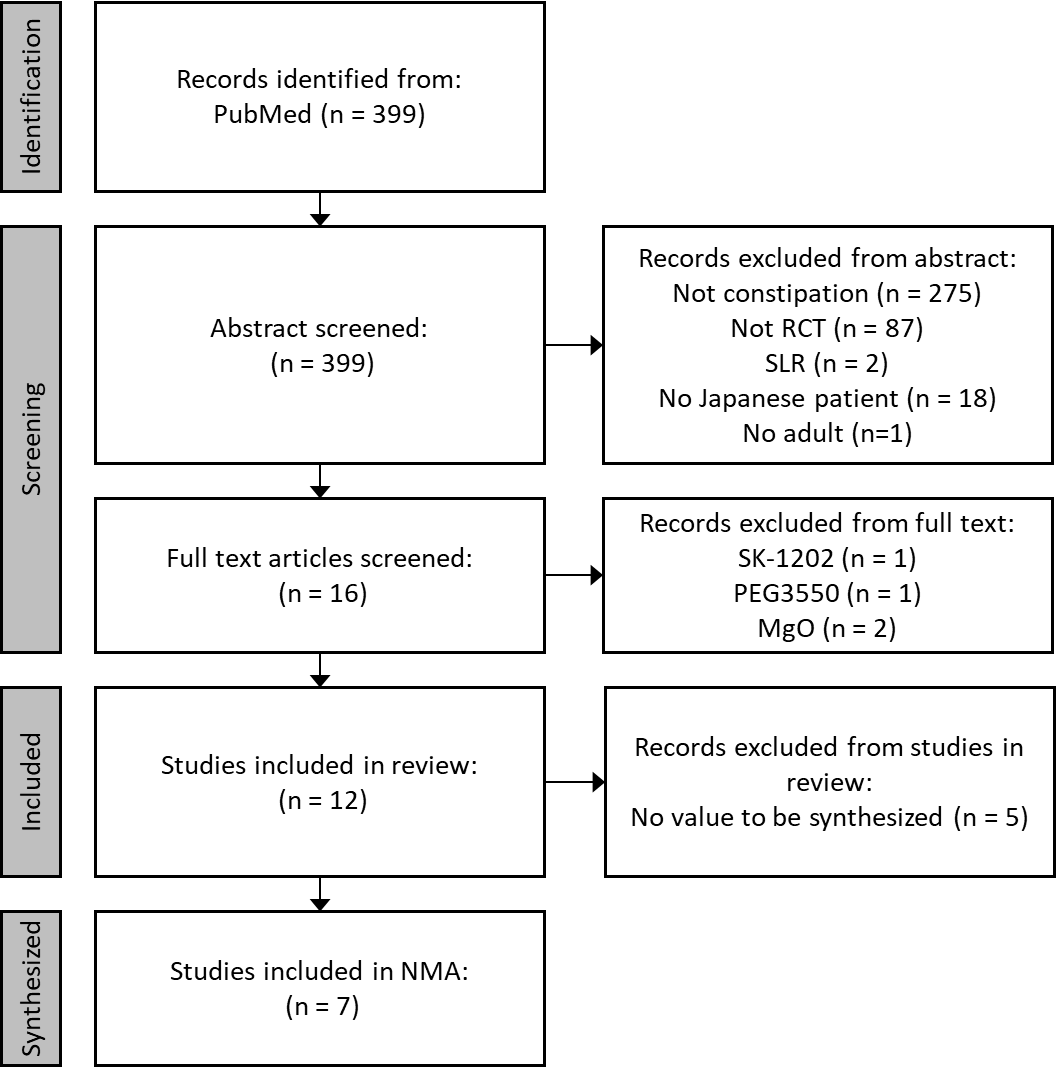


Figure S1. PRISMA Flow Diagram.

NMA, network meta-analysis; PRISMA, Preferred Reporting Items for Systematic reviews and Meta-Analyses; RCT, randomized controlled trial; SLR, systematic literature review.

Data were extracted by AS and reviewed for accuracy by AU and TO. The same information as the previous study [1] was extracted from each eligible article: first author, study year, number of cases, number of controls, age, number of women and men, drug studied, dosage form of the drug, number of measurement days, follow-up period, and proportion of patients with irritable bowel syndrome constipation. Mean change in weekly SBMs was also extracted as an outcome. If standard deviations (SDs) were not specified for the means, we extracted measures that enabled us to estimate the SD (e.g., standard error of the mean or confidence interval). If outcome measurements were reported at multiple times, we used the data from the time closest to that most often reported across the RCTs. If numerical data were not specified in the text or tables, we obtained them from the figures. Finally, we confirmed that the extracted values were same as those in the previous study [1] (Table S2). The risk of bias summary and asymmetric funnel plot of included studies are reported in the previous NMA study [1].

**Table S2. Extracted information for NMA**

| Study ID | Loc | Diagnosis | IBS-C | Age  (mean ± SD） | P of  females | Trial stage | Intervention | N | Change in SBM  (per week) (week 1) | | Ref no. |
| --- | --- | --- | --- | --- | --- | --- | --- | --- | --- | --- | --- |
|  |  |  |  |  |  |  |  |  | Mean | SD |  |
| Kumagai Y  2018 | Japan | <3 SBMs per week,  Rome III diagnostic criteria for  functional constipation | Included | 35.4 ± 10.8 | Not  reported | I | Placebo | 10 | 1.40 | 1.80 | 2 |
|  |  |  |  |  |  |  | Elobixibat 2.5mg | 10 | 5.90 | 2.91 |  |
|  |  |  |  |  |  |  | Elobixibat 5mg | 10 | 8.50 | 2.50 |  |
|  |  |  |  |  |  |  | Elobixibat 10mg | 10 | 9.20 | 4.71 |  |
|  |  |  |  |  |  |  | Elobixibat 15mg | 9 | 9.50 | 4.29 |  |
|  |  |  |  |  |  |  | Elobixibat 20mg | 9 | 14.70 | 3.69 |  |
| Nakajima A  2018 | Japan | <3 SBMs per week,  Rome III diagnostic criteria for  functional constipation | Included | 43.4 ± 13.3 | 82.6% | III | Placebo | 63 | 1.70 | 1.59 | 3 |
|  |  |  |  |  |  |  | Elobixibat 10mg | 69 | 6.40 | 4.98 |  |
| Nakajima A  2018 | Japan | <3 SBMs per week,  Rome III diagnostic criteria for  functional constipation | Included | 44.6 ± 12.8 | 87.7% | II | Placebo | 40 | 2.60 | 2.91 | 4 |
|  |  |  |  |  |  |  | Elobixibat 5mg | 43 | 3.50 | 1.03 |  |
|  |  |  |  |  |  |  | Elobixibat 10mg | 39 | 5.70 | 4.18 |  |
|  |  |  |  |  |  |  | Elobixibat 15mg | 41 | 5.60 | 3.52 |  |
| Fukudo S  2019 | Japan | <3 SBMs per week,  Rome III diagnostic criteria for  functional constipation | Not  included | 42.7 ± 11.9 | 82.3% | III | Placebo | 88 | 1.48 | 3.00 | 5 |
|  |  |  |  |  |  |  | Linaclotide 0.5mg | 91 | 4.02 | 2.96 |  |
| Fukudo S  2018 | Japan | <3 SBMs per week,  Rome III diagnostic criteria for  functional constipation | Not  included | 41.6 ± 11.4 | 83.2% | II | Placebo | 80 | 1.91 | 3.13 | 6 |
|  |  |  |  |  |  |  | Linaclotide 0.0625mg | 82 | 3.89 | 3.17 |  |
|  |  |  |  |  |  |  | Linaclotide 0.125mg | 71 | 3.11 | 2.93 |  |
|  |  |  |  |  |  |  | Linaclotide 0.25mg | 72 | 3.87 | 2.97 |  |
|  |  |  |  |  |  |  | Linaclotide 0.5mg | 76 | 3.85 | 3.05 |  |
| Fukudo S  2015 | Japan | <3 SBMs per week,  Rome III diagnostic criteria for  functional constipation | Included | 42.1 ± 15.3 | 87.9% | III | Placebo | 62 | 1.26 | 1.81 | 7 |
|  |  |  |  |  |  |  | Lubiprostone 48μg | 62 | 3.66 | 2.83 |  |
| Fukudo S  2011 | Japan | <3 SBMs per week,  Rome III diagnostic criteria for  functional constipation | Included | 39.5 ± 11.7 | 90.6% | II | Placebo | 42 | 1.50 | 2.59 | 8 |
|  |  |  |  |  |  |  | Lubiprostone 16μg | 41 | 2.30 | 2.56 |  |
|  |  |  |  |  |  |  | Lubiprostone 32μg | 43 | 3.50 | 3.28 |  |
|  |  |  |  |  |  |  | Lubiprostone 48μg | 44 | 6.80 | 7.30 |  |

IBS-C, irritable bowel syndrome with constipation; Loc, location; N, number; NMA, network meta-analysis; P, proportion; Ref no., reference number; SBM, spontaneous bowel movement; SD, standard deviation.

We conducted a network meta-analysis using the same method as the previous study, as follows. Data were abstracted and analyzed using R (version 4.3.0, R Foundation for Statistical Computing). The level of heterogeneity was similar to that of the previous study [1]. The traditional pairwise meta-analyses for every treatment (i.e., direct comparisons), using the DerSimonian-Laird random-effects model (“metafor” package in R), showed significant heterogeneity (I2 = 88.91%; P = 0.003). The funnel plot appeared to be asymmetric (Figure S2).


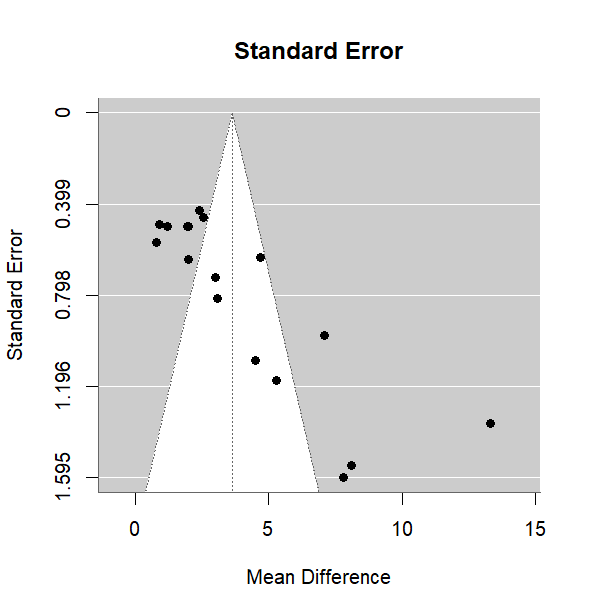


Figure S2 The funnel plot on change in weekly spontaneous bowel movements.

The Bayesian hierarchical network meta-analysis with a random-effects model used noninformative priors and a Markov chain Monte Carlo (MCMC) simulation (“gemtc” package version 0.8-2, which recalls JAGS [version 4.3.0, MRC Biostatistics Unit, Cambridge, UK] in R for MCMC sampling). We used 4 parallel chains and ran 20000 simulations to obtain model parameters after 5000 burn-in samples for each chain.

The estimated mean differences were 5.003 for elobixibat 10 mg, 2.239 for linaclotide 0.5 mg, and 3.560 for lubiprostone 48 μg (Figure S3).


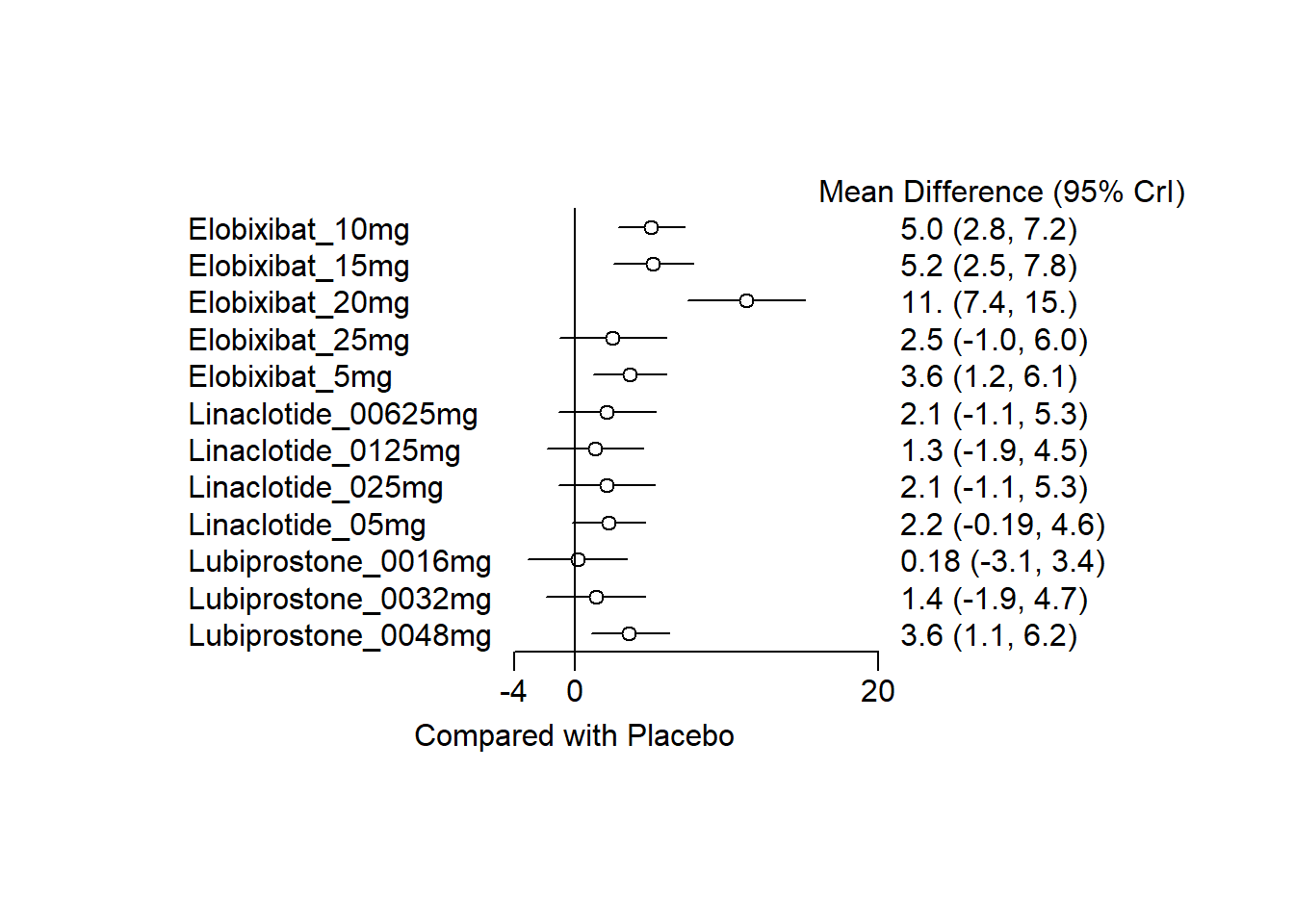


Figure S3. Forest plot of random-effect meta-analysis results.

**Sensitivity Analysis**

The range of the deterministic sensitivity analysis and distribution of the probabilistic sensitivity analysis are shown in Table S3.

| Parameters |  |  | Value | DSA range (Lower - Upper) | PSA distribution |
| --- | --- | --- | --- | --- | --- |
| Transition probability |  |  |  |  | Dirichlet |
| Risk ratio to Elobixibat |  | Linaclotide | 0.716 | 0.572 - 0.859 | Lognormal |
|  |  | Lubiprostone | 0.883 | 0.706 - 1.059 | Lognormal |
| QoL |  | Improved state | 0.145 | 0.116 - 0.174 | Beta |
|  |  | Unimproved state | 0.126 | 0.101 - 0.151 | Beta |
| Costs (JPY) | Drug price* | Elobixibat | 4,715 | 3,772 - 5,658 | Gamma |
|  |  | Linaclotide | 3,870 | 3,096 - 4,644 | Gamma |
|  |  | Lubiprostone | 5,600 | 4,480 - 6,720 | Gamma |
|  | Medical (disimpaction + enema) |  | 1,633 | 1,307 - 1,960 | Gamma |
|  | Caregiving | Improved state | 727 | 0,581 - 0,872 | Gamma |
|  |  | Unimproved state | 1,049 | 0,839 - 1,259 | Gamma |
|  | Productivity loss | Improved state | 100,910 | 80,728 - 121,092 | Gamma |
|  |  | Unimproved state | 59,003 | 47,202 - 70,803 | Gamma |

Table S3. Parameters used in the sensitivity analysis

DSA, deterministic sensitivity analysis; JPY, Japanese yen; PSA, probabilistic sensitivity analysis; QoL, quality of life.

* The unit costs for f elobixibat 10 mg, linaclotide 0.5 mg, and lubiprostone 48 μg were calculated based on the cost per day from the unit price, listed as of the fiscal year 2024

The proportion of patients in the improved state decreased with time, and the QoL accumulated per cycle decreased (Figure S4). We reported QoL results divided by 26, 65, and 130 (the number of cycles in the 2-, 5-, and 10-year time horizons, respectively), which indicate that a longer time horizon led to a smaller difference in QoL between drugs.


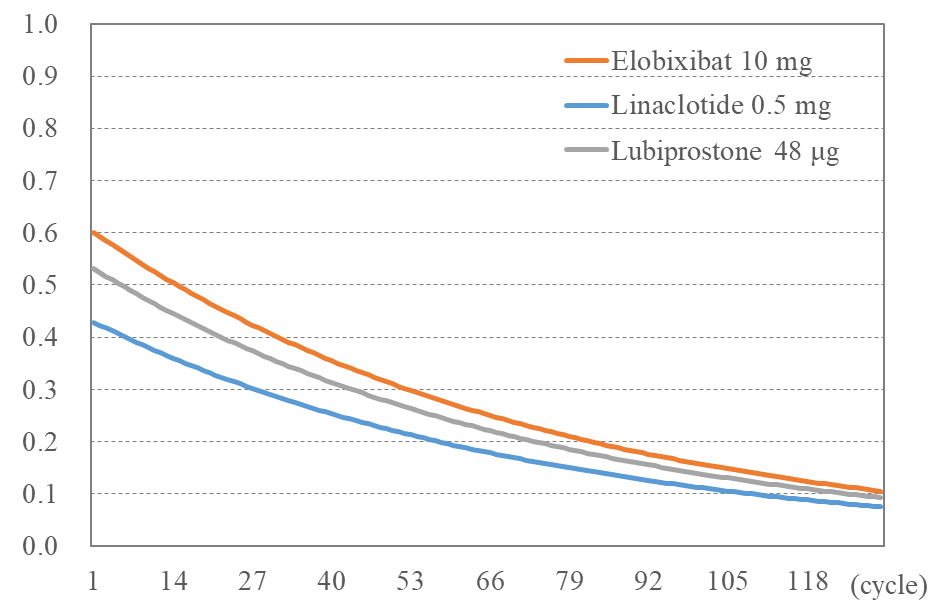


Figure S4. Change in the proportion of patients in the improved state

**References**

1. Nakajima A, Shoji A, Kokubo K, Igarashi A. A Systematic Review and Network Meta-Analysis on the Efficacy of Medications in the Treatment of Chronic Idiopathic Constipation in Japan. Gastroenterol Res Pract. 2021;2021(1):1-13. <https://doi.org/10.1155/2021/5534687>

2. Kumagai Y, Amano H, Sasaki Y, Nakagawa C, Maeda M, Oikawa I, et al. Effect of single and multiple doses of elobixibat, an ileal bile acid transporter inhibitor, on chronic constipation: A randomized controlled trial. Brit J Clin Pharmacol. 2018;84(10):2393-404. <https://doi.org/10.1111/bcp.13698>

3. Nakajima A, Seki M, Taniguchi S, Ohta A, Gillberg P, Mattsson JP, et al. Safety and efficacy of elobixibat for chronic constipation: results from a randomised, double-blind, placebo-controlled, phase 3 trial and an open-label, single-arm, phase 3 trial. Lancet Gastroenterol Hepatol. 2018;3(8):537-47. <https://doi.org/10.1016/s2468-1253(18)30123-7>

4. Nakajima A, Seki M, Taniguchi S. Determining an optimal clinical dose of elobixibat, a novel inhibitor of the ileal bile acid transporter, in Japanese patients with chronic constipation: a phase II, multicenter, double-blind, placebo-controlled randomized clinical trial. J Gastroenterol. 2018;53(4):525-34. <https://doi.org/10.1007/s00535-017-1383-5>

5. Fukudo S, Miwa H, Nakajima A, Kinoshita Y, Kosako M, Hayashi K, et al. High‐dose linaclotide is effective and safe in patients with chronic constipation: A phase III randomized, double‐blind, placebo‐controlled study with a long‐term open‐label extension study in Japan. Neurogastroenterol Motil. 2019;31(1):e13487. <https://doi.org/10.1111/nmo.13487>

6. Fukudo S, Miwa H, Nakajima A, Kinoshita Y, Kosako M, Nakagawa A, et al. Dose‐finding study of linaclotide in Japanese patients with chronic constipation: A phase II randomized, double‐blind, and placebo‐controlled study. Neurogastroenterol Motil. 2018;30(12):e13442. <https://doi.org/10.1111/nmo.13442>

7. Fukudo S, Hongo M, Kaneko H, Takano M, Ueno R. Lubiprostone Increases Spontaneous Bowel Movement Frequency and Quality of Life in Patients With Chronic Idiopathic Constipation. Clin Gastroenterol Hepatol. 2015;13(2):294-301.e5. <https://doi.org/10.1016/j.cgh.2014.08.026>

8. Fukudo S, Hongo M, Kaneko H, Ueno R. Efficacy and safety of oral lubiprostone in constipated patients with or without irritable bowel syndrome: a randomized, placebo-controlled and dose-finding study. Neurogastroenterol Motil. 2011;23(6):544-e205. <https://doi.org/10.1111/j.1365-2982.2011.01668.x>
